# Inequalities in the Global Burden of Chronic Kidney Disease Due to Type 2 Diabetes Mellitus: An Analysis of Trends from 1990 to 2019

**DOI:** 10.3390/ijerph18094723

**Published:** 2021-04-28

**Authors:** Nóra Kovács, Attila Nagy, Viktor Dombrádi, Klára Bíró

**Affiliations:** 1 Department of Public Health and Epidemiology, Faculty of Medicine, University of Debrecen, 4028 Debrecen, Hungary; kovacs.nora@med.unideb.hu; 2 Faculty of Public Health, University of Debrecen, 4028 Debrecen, Hungary; nagy.attila@sph.unideb.hu; 3 Health Services Management Training Centre, Faculty of Health and Public Administration, Semmelweis University, 1125 Budapest, Hungary; dombradi.viktor@emk.semmelweis.hu; 4 Department of Health Systems Management and Quality Management for Health Care, Faculty of Public Health, University of Debrecen, 4032 Debrecen, Hungary

**Keywords:** disease burden, chronic kidney disease, global burden of disease, inequalities, socioeconomic development

## Abstract

The prevalence of type 2 diabetes mellitus (T2DM) and the burden of complications are increasing worldwide. Chronic kidney disease (CKD) is one serious complication. Our aim was to investigate the trends and inequalities of the burden of CKD due to T2DM between 1990 and 2019. Data were obtained from the Global Health Data Exchange database. Age-standardized incidence, mortality, and DALYs rates of CKD were used to estimate the disease burden across the Human Development Index (HDI). Joinpoint regression was performed to assess changes in trend, and the Gini coefficient was used to assess health inequality. A higher incidence was observed in more developed countries (*p* < 0.001), while higher mortality and DALYs rates were experienced in low and middle HDI countries in 2019 (*p* < 0.001). The trend of incidence has increased since 1990 (AAPC: 0.9–1.5%), while slight decrease was observed in low HDI countries in mortality (APC: −0.1%) and DALYs (APC: −0.2%). The Gini coefficients of CKD incidence decreased from 0.25 in 2006 to 0.23 in 2019. The socioeconomic development was associated with disease burden. Our findings indicate that awareness of complications should be improved in countries with high incidence, and cost-effective preventive, diagnostic, and therapeutic tools are necessary to implement in less developed regions.

## 1. Introduction

According to the latest estimations of the International Diabetes Federation (IDF), around 463 million 20–79 years old adults have diabetes mellitus (DM) worldwide. The burden of this disease is enormous; around 10% of global health expenditure (USD 760 billion) is spent on diabetes. As for geographic patterns, around 79% of all diabetes are from low- and middle-income countries [1]. Besides the significant increase in prevalence, the comorbidities and complications are getting higher as well.

Chronic kidney disease (CKD) is one of the most dangerous complications, and it is often asymptomatic, with rapid progression. Nephropathy is frequently associated with type 2 diabetes mellitus (T2DM), and it is considered the leading cause of both CKD and end-stage renal disease [2,3]. Additionally, the prevalence of renal failure is increasing with T2DM [4]. However, diabetic nephropathy may be underestimated as an underlying cause of renal disease because diabetes can remain undetected for many years [5]. The results of the Global Burden of Disease Study in 2017 stated that the incidence, prevalence, and mortality of CKD in T2DM patients has increased to 31.6%, 27.0%, and 34.0%, respectively [3,4]. However, the burden associated with CKD is not homogenous, as the low- and middle-income countries are disproportionately affected [6]. Therefore, the progressive rise in CKD prevalence puts a substantial burden on all, especially less developed countries with a weak healthcare system, without available and affordable care for the disease [7,8]. Besides national disparities, patients with lower socioeconomic status are more likely to experience a wide range of obstacles to diabetes treatment, such as access to healthcare services, cost, poor health, and greater disability [9,10]. Furthermore, microvascular and macrovascular diabetes complications may be more common in T2DM patients with the lowest socioeconomic status at both individual or geographical level [11].

Therefore, quantifying the number of people living with T2DM and its complications is crucial for policymakers and healthcare providers to optimally allocate limited resources, develop preventive measures, and determine the direction of public health interventions aimed at reducing disease burden.

Taking these into consideration, the aim of the study was to explore the trend and variations in incidence, mortality, and burden of chronic kidney disease caused by T2DM globally, according to the socioeconomic level between 1990 and 2019.

## 2. Materials and Methods

### 2.1. Study Design

The annual incidence, mortality, and disability-adjusted life years (DALYs) rate for chronic kidney disease due to T2DM were estimated by the Global Burden of Disease (GBD) 2019 study. We obtained the data over 1990–2019 from the publicly available Global Health Data Exchange database (http://ghdx.healthdata.org/gbd-results-tool, accessed on 13 March 2021) [12]. The GBD 2019 study provides comprehensive perspectives for the health burden of 369 diseases and injuries across 204 countries and territories, for age groups, sexes, countries, regions. The data is available on global and national levels annually between 1990 and 2019 [13].

Data from all the 204 countries were collected using the GBD 2019 database. The relevant burden of CKD was expressed in terms of incidence, mortality, and DALYs. Accordingly, the following data were collected regarding the chronic kidney disease caused by T2DM globally and at the national level: age-standardized incidence rate (ASIR), age-standardized mortality rate (ASMR), and age-standardized DALYs rate from 1990 to 2019. The rates of CKD are referring to the rate per 100,000 population. We used age-standardized rates in the analyses due to the rapid population growth and changing age composition. The DALY was used to measure the health burden of chronic kidney disease caused by T2DM, which combines the years of life lost (YLL) and years lost due to disability (YLD) [13,14]. DALYs considers both the length and quality of life and is used in many countries to inform health policies, international organizations, and health system planning. The detailed methods for calculating age-standardized incidence and mortality rates and DALYs can be found in GBD publications [15].

### 2.2. Socioeconomic Status

The Human Development Index (HDI) was used to point out the national socioeconomic status (SES), which is a comprehensive indicator reflecting human development in health, education, and income dimensions. The HDI is calculated based on four components: life expectancy at birth, mean years of schooling, mean and expected years of school education, and gross national income (GNI) per capita [16]. We collected HDI data for each country from the United Nations Development Programme (UNDP; http://hdr.undp.org/en/data, accessed on 13 March 2021). The HDI ranges from 0 to 1, with a higher value indicating a higher level of socioeconomic development. We categorized the countries into four groups in 2019 according to the value of HDI and based on the UNDP suggestion: very high human development (HDI ≥ 0.804), high (0.703 ≤ HDI < 0.804), medium (0.554 ≤ HDI < 0.703), and low (HDI < 0.554). The burden of CKD was compared across each group of HDI categories. We included 187 countries with available HDI data in the analyses, estimating the current disease burden across socioeconomic status in 2019.

### 2.3. Gini Coefficient

We used the Gini coefficient to assess the trend of CKD-associated health inequality between countries over time from 1990 to 2019. The Gini coefficient (or Gini index) was calculated based on the Lorenz curve and to measure inequalities of a nation in incidence, mortality, and DALYs rates. A Lorenz curve demonstrates the cumulative distribution of health in a nation ranked by health, and the Gini coefficient measures the deviation from an equal distribution. The coefficient ranges from 0 (perfect equality) and 1 (total inequality) [17,18]. The annual ASIR, ASMR, and age-standardized DALYs of CKD for each country were used to calculate the Gini coefficients.

### 2.4. Statistical Analysis

We performed a Kruskal–Wallis test to evaluate differences of age-standardized incidence, mortality, and DALYs across HDI categories, followed by a Dunn test for post hoc multiple comparisons of groups. The unit of analysis was country or territory. Linear regression analysis and scatter plots were used to investigate and show the relationship between age-standardized measures of CKD and the level of socioeconomic development. A joinpoint regression analysis was performed to detect significant changes in trends in terms of the ASIR, ASMR, and age-standardized DALYs rates during 1990–2019 [19]. The maximum number of joinpoints was set to three, and a significant change in the rates over time was assessed by the permutation test. Each p-value given by the permutation tests was estimated by Monte Carlo methods [19]. The annual percent change (APC) for the segments and average annual percent change (AAPC) as a weighted average of the APCs for the overall period were computed. The APC is used to characterize trends in rates over time by a change at a constant percentage of the previous year’s rate, while the AAPC expresses the average APCs over a period of many years. All joinpoint analyses were performed using Joinpoint Statistical Software (Joinpoint Regression Program, Version 4.8.0.1-April 2020, National Cancer Institute, Bethesda, MD, USA; Statistical Methodology and Applications Branch, Surveillance Research Program, National Cancer Institute) [20].

Statistical analyses were performed using STATA IC version 13.0 software (Stata Corp., College Station, TX, USA). The Gini coefficient values were computed using the AINEQUAL16 modules of Stata 13.0 software (Stata Corp., College Station, TX, USA). *p*-value < 0.05 was considered as statistically significant.

## 3. Results

### 3.1. Trends in Global Burden of CKD over Time

**Figure 1:**
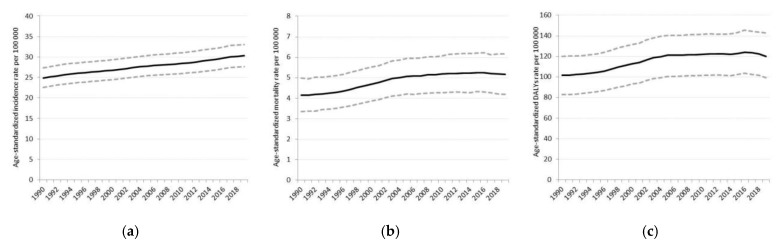
Time trends in (**a**) age-standardized incidence rates, (**b**) age-standardized mortality rates, and (**c**) age-standardized DALYs rates of CKD from 1990–2019. Dashed lines show the 95% uncertainty intervals.

To describe the change in the overall burden of CKD, trends were presented from 1990 to 2019. The ASIR has increased continuously from 24.9 (95% uncertainty interval (UI): 22.6–27.4) in 1990 to 30.3 per 100,000 (95% UI: 27.6–33.1) in 2019. The ASMR has changed from 4.1 (95% UI: 3.3–4.9) to 5.2 per 100,000 (95% UI: 4.2–6.2) during the period. The trend of age-standardized DALYs was similar to the mortality trend; the rate increased to 120.2 (95% UI: 99.2–142.9) from 101.7 per 100,000 (95% UI: 82.9–120.1). Both ASMR and DALYs rates showed a slight decrease in the most recent years. (Figure 1a–c).

### 3.2. Current Disease Burden of CKD According to HDI

**Figure 2:**
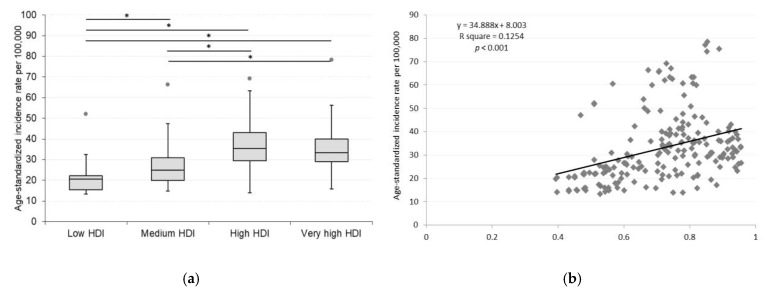

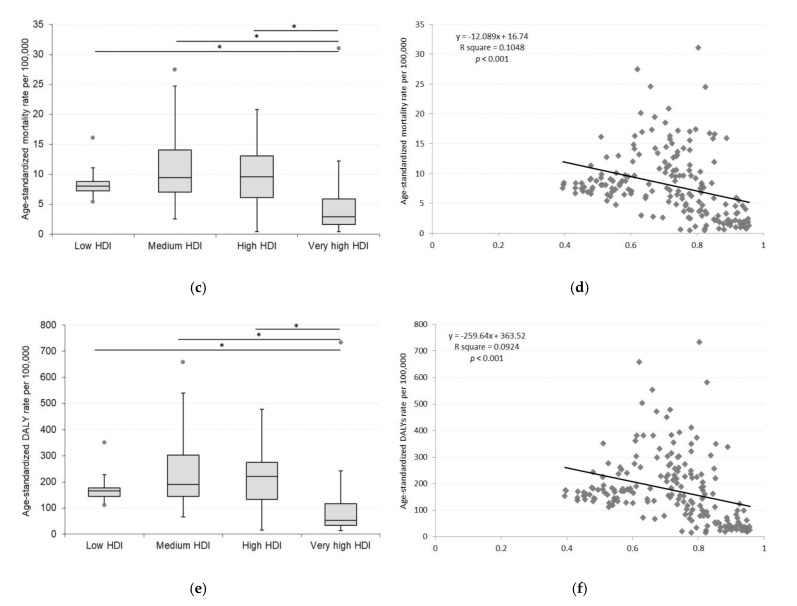
Association between socioeconomic development and (**a**,**b**) ASIR, (**c**,**d**) ASMR, and (**e**,**f**) age-standardized DALYs rate of CKD in 2019. * *p* < 0.05.

The distribution of age-standardized rates of CKD according to HDI groups in 2019 is presented in Figure 1a–c. Of the original 204 countries, the HDI data was available for 187 countries in 2019, including 33 low, 37 medium, 53 high, and 64 very high HDI countries. The age-standardized incidence, mortality, and DALYs rates of CKD varied significantly across countries with different human development levels according to the Kruskal–Wallis test (*p* = 0.001). According to pairwise comparison, ASIR in low HDI countries differed significantly from medium (*p* = 0.007), high (*p* < 0.001) and very high (*p* < 0.001) HDI countries. In the case of ASMR and age-standardized DALYs rates, only very high HDI countries showed a significant difference from low (*p* < 0.001), medium (*p* < 0.001), and high HDI countries (*p* < 0.001). (Figure 2a,c,e) We observed a positive relationship between the ASIR and HDI values (*p* < 0.001). The countries with a higher level of social development are experienced higher incidence. In contrast, negative association was determined between ASMR (*p* < 0.001) and DALYs rates (*p* < 0.001) and HDI values. (Figure 2b,d,f).

### 3.3. Trends in CKD Incidence, Mortality, and DALYs Rates Across HDI Groups Over Time

The joinpoint regression analysis showed a rapid, significant increasing trend in ASIR across all HDI groups. The highest increase was shown by the high HDI group during the time period (AAPC: 1.5%), and a high increase was observed in low (2015–2019, APC: 1.9%) and middle HDI (2017–2019, APC: 1.4%) countries in recent years. The trend was not homogenous in ASMR and DALYs rates across HDI categories. In the low HDI countries, the ASMR showed a quite continuous significant decrease (AAPC: −0.1%), and a similar trend was also observed in the case of DALYs rates (AAPC: −0.2%). In the middle HDI group, a declining trend was observed for the ASMR from 2014 by an average decrease of 0.2% per year and for DALYs rates from 2015 by an average decrease of 0.5% per year. In high HDI countries, the ASMR and DALYs rates also decreased from 2016 to 2019 (ASMR, APC: −0.4%; DALYs rate, APC: −0.2%). A similar pattern of change to middle and high HDI countries was observed in the most developed countries. The mortality rates (2010–2019, APC: −0.8%) and disease burden (2009–2019, APC: −0.8%) has also decreased in the last decade in very high HDI countries; however, the rate of change was greater. (Table 1).

### 3.4. Global Health Inequality Related to CKD

**Figure 3:**
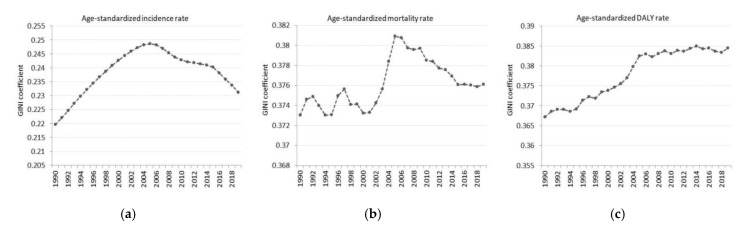
Trends in the Gini coefficients calculated based on (**a**) age-standardized incidence rates, (**b**) age-standardized mortality rates, and (**c**) age-standardized DALY rates across 204 countries globally between 1990 and 2019.

According to the Gini coefficient, unequal geographical health inequality between countries was observed. The inequality related to incidence rate increased between 1990 (0.22) and 2005 (0.25), followed by a strong decreasing trend, which was observed from 2006 (0.25) to 2019 (0.23), despite the significant decrease observed in the age-standardized incidence rate (Figure 3a). Regarding ASMR, inequality increased from a starting value of 0.37 in 1990 until 2006 (0.38), when it started to decrease, and the value of the indicator was 0.37 in the most recent year (Figure 3b). Finally, increasing health inequality disparities were observed according to CKD caused DALYs rates between countries; therefore, the inequality for DALYs continuously increased from 0.37 in 1990 to 0.38 in 2019, respectively. (Figure 3c).

## 4. Discussion

In this study, we investigated the trends and variations of incidence, mortality, and disease burden of chronic kidney disease due to T2DM globally. In order to provide a more detailed assessment across countries, we used age-standardized measures for national comparison, according to socioeconomic development levels expressed in HDI. The HDI, which is a standard socioeconomic indicator, summarizes the three key dimensions of human development (life expectancy at birth, mean and expected years of education, and GNI per capita) [16] and allows comparison across countries by different levels of human development. Socioeconomic indicators were also used by others to express the relationship between socioeconomic determinants and national disease burdens [21,22,23].

The results showed that socioeconomic development was associated with ASIR, ASMR, and DALYs rates. A higher incidence of CKD was concentrated in countries with a higher level of development in 2019, while lower mortality and DALYs rates were reported in more developed countries. The inequalities in deaths and disease burden can be attributed to the higher quality of healthcare resulted in better clinical outcomes in more developed countries, while in developing countries, a considerable number of patients with diabetes do not meet the treatment targets. Moreover, the high cost and non-availability of drugs and treatment and the lack of consistent guidelines for diabetes prevention and management are further challenges in many developing countries [24]. The proper diagnosis and better access to health care are reflected in higher incidence in countries with higher HDI values. Further increase in the CKD incidence is expected parallel with the diabetes incidence, as a result of western lifestyle and associated obesity in more industrialized countries [25]. The lack of appropriate diagnostic tools may lead to missed or misdiagnosed diabetes in developing countries. In addition, the preparedness of human resources is also crucial. A low level of knowledge can result in poor disease management and the occurrence of complications [26].

The results of the study also highlight that although the health inequalities of overall CKD burden has improved during the past decades, our results suggest that discrepancies in the global burden had not been eliminated yet, considering that inequality by socioeconomic status remains a public health challenge for reducing the global burden of CKD.

The ASIR has continuously increased since 1990 globally, which may be explained by better detection of diabetes and extended life expectancy. The decreasing trend of mortality and DALYs rates in recent years in the middle HDI group appeared after a rapid growth, which suggests that countries with medium socioeconomic levels have undergone social and economic transition the past few decades, which poses a challenge to the healthcare system [13].

Previous studies showed that the socioeconomic level and the regional deprivation are strongly associated with diabetes prevalence. Besides the duration of the disease, the socioeconomic level of the patients also has a significant impact on the occurrence of diabetic complications. Poor availability of healthcare services, more difficult access to healthcare resources, higher costs associated with treating diabetes complications, inadequate self-efficacy, and inability to meet HbA1c targets are common features of the more disadvantaged communities, which tend to explain the correlation between low SES and higher risk of diabetes complications. Moreover, in certain nations, the provision of health insurance is still a significant impediment to achieve improved health [11]. According to the literature, lower SES and larger economic distress have a negative impact on glycemic control as well as on kidney-related complications [11,27].

Although low SES is also associated with an increased risk of CKD independently of the presence of diabetes [28,29,30,31,32], low SES can affect the distribution of CKD in the community in a complex way. Variations in population may be due to a variety of factors as the demographic composition of the population, the existence of comorbidities (such as hypertension), health behavior, or healthcare system-related features (for example, access to care) [33].

This research also highlights the importance of preventing complications in T2DM patients. Beyond the socioeconomic factors, others, such as preventing T2DM and early medical interventions play an important role in the prevention of renal failures [2,7]. Raising awareness of maintaining a healthy lifestyle is also a critical aspect because patients from disadvantaged socioeconomic areas are less likely to practice healthy habits such as consuming fruits and vegetables regularly or quitting smoking [34].

Global concerns about the growing end-stage renal disease prevalence and the high burden of CKD around the world are attributed to the rising costs of therapies, and negative outcomes of CKD manifested in higher disease burden and mortality. Early detection and intervention services, which are considered to be the most cost-effective approaches, may help to avoid or at least prolong unfavorable health consequences of renal failures, and this requires a comprehensive knowledge of the disease burden and the associated risk factors in the population [8]. Given that the high economic costs associated with diabetes are expected to grow further, public health interventions should focus on advanced and effective approaches in the care of diabetes and its complications [24,35].

Further researches are needed to understand a broader context of determinants that are related to health burden of CKD in population. In addition, follow-up studies would allow exploring factors other than socioeconomic determinants in details that influence the development and outcome of complications in patients with T2DM.

### Strengths and Limitations

A strength of this study is the provision of comprehensive population-based estimates of data on patterns and trends of burden due to T2DM caused CKD globally and according to different development categories. The study period we analyzed was long, offering a comprehensive overview about disease burden of CKD. One of the limitations of our study is that the trend observed in joinpoint regression analysis may change by parameter settings and amount of data analyzed. The unit of analysis was countries; therefore, the distribution of data can be heterogeneous within countries and territories, which is covered by the nationally aggregated data. Data may show variations in some countries and regions due to the different way of data collection, ethnicity, lifestyle or access to healthcare. The health burden may be underestimated because of the hidden morbidity of CKD and diabetes, particularly in less developed countries. The DALYs can be used to evaluate the disease burden across countries and over time, but it is calculated without taking into account the broader aspects and social impact of diseases [36]. Furthermore, the nature of CKDs is complex in terms of causes and possible clinical outcomes. Due to the aggregated dataset, relevant individual and clinical data could not be considered in our analyses, for example the onset and classification of diabetic nephropathy, data on renal function, and duration of diabetes. Nevertheless, despite these limitations, the databases and methodology used in this study were adequate enough to identify inequalities and trends regarding the global burden of CKD.

## 5. Conclusions

The current patterns and trends in CKD due to T2DM incidence, mortality, and DALYs rates correlated with the socioeconomic levels. Our findings highlight that physicians’ adherence to diabetes guidelines and awareness of complications should be reviewed, and if needed, improved, particularly in most developed countries where the incidence of CKD is high and increasing further. Furthermore, to avoid the premature deaths caused by CKD, policymakers should consider that reducing inequalities in the socioeconomic status can lead to a reduction in the risk of diabetes complications. As a result, there is a need for more accurate and cost-effective diagnostic tools and treatment for diabetes and its complications, especially in low- and middle-income countries where the healthcare resources are really scarce. In addition to early detection of diabetes and its complications, slowing the progression of CKD also has considerable economic benefit [8,37]. Patient education and training of medical professionals may also be considered as further inexpensive and effective ways of achieving better health outcomes.

## Figures and Tables

**Table 1:** Age-standardized CKD incidence, mortality, and DALYs rates in 1990, 2019, and joinpoint trend analysis between 1990 and 2019 by HDI settings.

**INCIDENCE**	**Trend 1**		**Trend 2**		**Trend 3**		**Trend 4**		**1990–2019**
	**Period**	**APC (%)**	**Period**	**APC (%)**	**Period**	**APC (%)**	**Period**	**APC (%)**	**AAPC (%)**
Low HDI	1990–1999	0.5 *	1999–2007	1.5 *	2007–2015	1.0 *	2015–2019	1.9 *	1.1 *
Middle HDI	1990–2002	1.3 *	2002–2014	1.4 *	2014–2017	1.7 *	2017–2019	1.4 *	1.4 *
High HDI	1990–1995	2.2 *	1995–2004	1.8 *	2004–2013	1.3 *	2013–2019	1.1 *	1.5 *
Very high HDI	1990–1993	1.5 *	1993–1999	1.3 *	1999–2004	1.1 *	2004–2019	0.6 *	0.9 *
**MORTALITY**	**Trend 1**		**Trend 2**		**Trend 3**		**Trend 4**		**1990–2019**
	**Period**	**APC (%)**	**Period**	**APC (%)**	**Period**	**APC (%)**	**Period**	**APC (%)**	**AAPC (%)**
Low HDI	1990–1995	−0.1	1995–1999	−0.4 *	1999–2003	0.0	2003–2019	−0.1 *	−0.1 *
Middle HDI	1990–2004	1.7 *	2004–2010	0.2 *	2010–2014	0.6 *	2014–2019	−0.2 *	0.9 *
High HDI	1990–1994	0.9 *	1994–2002	1.6 *	2002–2016	0.5 *	2016–2019	−0.4	0.8 *
Very high HDI	1990–1995	0.5	1995–2006	1.5 *	2006–2010	0.0	2010–2019	−0.8 *	0.4 *
**DALYs**	**Trend 1**		**Trend 2**		**Trend 3**		**Trend 4**		**1990–2019**
	**Period**	**APC (%)**	**Period**	**APC (%)**	**Period**	**APC (%)**	**Period**	**APC (%)**	**AAPC (%)**
Low HDI	1990–1995	−0.1	1995–2000	−0.5 *	2000–2003	0.1	2003–2019	−0.1 *	−0.2 *
Middle HDI	1990–2004	1.5 *	2004–2010	0.1	2010–2015	0.5 *	2015–2019	−0.5 *	0.7 *
High HDI	1990–1992	0.8	1992–2002	1.4 *	2002–2016	0.4 *	2016–2019	−0.2	0.7 *
Very high HDI	1990–1994	0.4	1994–2006	1.2 *	2006–2009	0.0	2009–2019	−0.8 *	0.3 *

* *p* < 0.05. Abbreviations: Human Development Index (HDI), annual percent change (APC), average annual percent change (AAPC).

## Data Availability

Publicly available datasets were analyzed in this study. This data can be found here: http://ghdx.healthdata.org/gbd-results-tool (accessed on 28 April 2021).
